# The Third Mobile Window Syndrome: A Clinical Spectrum of Different Anatomical Locations—Characterization, Therapeutic Response, and Implications in the Development of Endolymphatic Hydrops

**DOI:** 10.3390/jcm13237232

**Published:** 2024-11-28

**Authors:** Joan Lorente-Piera, Raquel Manrique-Huarte, Nicolás Pérez Fernández, Diego Calavia Gil, Marcos Jiménez Vázquez, Pablo Domínguez, Manuel Manrique

**Affiliations:** 1 Department of Otorhinolaryngology, Clínica Universidad de Navarra, 31008 Pamplona, Spain; 2 Department of Otorhinolaryngology, Clínica Universidad de Navarra, 28027 Madrid, Spain; 3 Department of Radiology, Clínica Universidad de Navarra, 31008 Pamplona, Spain

**Keywords:** Ménière’s disease, vertigo, third window syndrome

## Abstract

**Background/Objectives:** Multiple dehiscences of the otic capsule can exhibit behavior similar to Ménière’s disease, not only from a clinical perspective but also in the results of audiovestibular tests. The main objective of this study is to characterize third mobile window etiologies from an audiovestibular perspective, while also evaluating the therapeutic response to four different treatment protocols. Furthermore, we aim to explore a potential association with the development of radiologically defined endolymphatic hydrops (EH). **Methods**: This is a retrospective cohort study conducted from 2017 to 2024 at a tertiary-level otology and otoneurology unit. All patients underwent pure tone audiometry, vHIT, cVEMP, and oVEMP. Some of these patients, selected under rigorous inclusion criteria based on clinical and audiometric findings, were subjected to a 4-h delayed intravenous gadolinium-enhanced 3D-FLAIR MRI. **Results**: We obtained a sample of 86 patients, with a mean age of 52.2 ± 7.64 years: 62.76% were female (n = 54) and 37.21% were male (n = 32); 88.37% (n = 76) were diagnosed with superior semicircular canal dehiscence syndrome (SSCDS), while 11.62% (n = 10) had other forms of otic capsule dehiscence. The most common symptom observed was unsteadiness (44%). While surgery is the only curative treatment, other medical treatments, such as acetazolamide, also helped reduce symptoms such as autophony, falls, instability, and vertigo attacks, with a relative risk reduction (RRR) exceeding 75% (95% CI, *p* < 0.05). The results of the MRI in EH sequences indicate that 7.89% of the patients diagnosed with SSCDS also developed radiological EH, compared to 40.00% of the patients with other otic capsule dehiscences, a difference that was statistically significant (*p* = 0.0029. **Conclusions**: Otic capsule dehiscences are relatively unknown conditions that require clinical diagnosis. Although VEMP testing is useful, imaging studies are necessary to localize and characterize the defect, most commonly found in the superior semicircular canal. We should consider these dehiscences in cases where there is a suspicion of EH development. Further research, including in vivo neuroimaging studies using hydrops sequences, is required to better understand their relationship to potential Ménière’s disease.

## 1. Introduction

In many otoneurology consultations, diagnostic challenges arise due to clinical syndromes and pathologies presenting with a combination of ear fullness, episodes of vertigo, and associated hearing loss. After ruling out more common conditions like benign paroxysmal positional vertigo, vestibular neuritis, or vestibular migraine, it becomes crucial to differentiate these from entities that can exhibit Ménière-like symptoms.

In 2015, López-Escamez et al. [1] aimed to document a consensus through a specialized committee report, listing various conditions requiring differential diagnosis from Ménière’s disease (MD). These include superior semicircular canal dehiscence syndrome (SSCDS), perilymphatic fistula, and enlarged vestibular aqueduct, categorized under the term of third mobile window phenomena. Thus, the term third window is used to describe pathological conditions in which an additional opening to the round and oval windows appears in the inner ear, disrupting the normal dynamics of fluids and sound transmission [2].

Since SSDCS was first described, this finding, often occurring incidentally while investigating possible cochleovestibular syndromes, there has been a significant increase in literature describing cases compatible with third mobile window clinical presentation. In fact, from 2021, SSCDS received defined diagnostic criteria [3].

Other conditions with similar clinical profiles have since emerged, necessitating their consideration in the differential diagnosis of endolymphatic hydrops (EH) and other third mobile window etiologies [4,5,6]. In 2008, Merchant and Rosowski [7] proposed a general hypothesis to explain the underlying mechanism of hearing loss associated with these abnormalities. Typically, sound propagation occurs through the oval and round windows, which act as fluid interfaces between the air in the middle ear and the perilymphatic spaces of the inner ear. Several factors can create additional defects in the bony labyrinth, leading to the emergence of third hydrodynamic windows. These defects can be responsible for symptoms and may even alter fluid dynamics in the inner ear, potentially resulting in radiologically observable EH [8].

The primary aim of this study is to characterize the audiovestibular manifestations of third mobile window syndromes, with a specific focus on different types of otic capsule dehiscences, including but not limited to SSCDS. Additionally, the study seeks to evaluate the effectiveness of four distinct treatment protocols for managing these conditions. A secondary objective is to investigate the potential association between these dehiscences and the development of radiologically detectable EH, using targeted MRI sequences.

## 2. Materials and Methods

### 2.1. Study Design

This is a retrospective, multicenter cohort study including 86 patients attended and followed up in the otology and otoneurology units of two tertiary care centers between 2017 and 2024

### 2.2. Patient Selection

Patients of all ages were included in the study if they were diagnosed with third mobile window syndromes, identified either through imaging studies utilizing a high-resolution CT scan or based on the diagnostic criteria established by Ward et al. [3] for SSCDS. Eligible participants presented with auditory symptoms, such as hearing loss, autophony, or tinnitus, and/or vestibular symptoms, including vertigo, imbalance, or Hennebert or Tulio sign, that could not be attributed to other underlying conditions. Diagnosis was confirmed in all cases through detailed high-resolution computed tomography, which documented the presence of otic capsule dehiscences, including but not limited to the superior semicircular canal.

Patients were included only if they had comprehensive follow-up records and well-documented outcomes, both before and after treatment, with management tailored to their specific etiologies and clinical characteristics. Informed consent was obtained from all participants, ensuring their voluntary agreement to take part in the study in full compliance with the ethical principles outlined in the 1975 Declaration of Helsinki [9].

### 2.3. Examination and Complementary Tests

All patients underwent a physical examination, including otoscopy and an otoneurological examination using a videonystagmography (VNG) system (VideoFrenzel Interacoustics VF505m, Assens, Denmark). The audiovestibular tests included pure tone audiometry (PTA) (AC40, Interacoustics), vestibular evoked myogenic potentials (VEMP, Eclipse Interacoustics, Assens, Denmark), video head impulse test (vHIT, ICS Impulse GN Otometrics^®^ Natus Medical, Taastrup, Denmark), and computed tomography (CT) scans. These tests were conducted at the time of diagnosis, with VEMPs, PTA, and vHIT repeated during follow-up treatment.

- Audiological evaluation: Findings were reported in terms of pure tone average (PTA), including pre- and post-treatment PTA and gain, measured in decibels at 500, 1000, 2000, 4000, and 6000 Hz (AC 40, Interacoustics AS, Assens, Denmark). The degree of hearing loss was classified according to the criteria of the Bureau International d’Audiophonologie (BIAP) [10].
- Vestibular evaluation: vHIT was used to analyze the gain of the vestibulo–ocular reflex, considering values below 0.8 as abnormal and evaluating the presence of corrective saccades in each of the three semicircular canals in both ears [11]. For VEMP, both cervical (cVEMP) and ocular (oVEMP) tests were conducted. An abnormal vestibular function was defined as a VEMP response in both ears with an interaural asymmetry ratio (IAAR %) exceeding 40%. Asymmetry ratios were analyzed for air-conducted stimulation at 0.5 kHz, 1 kHz, and 4 kHz, with the acoustic stimulus intensity set at 97 dB normalized [12].
- Imaging studies: Thin-section helical CT scans (SOMATOM CT Scanner Dual Source and Single Source, Siemens Healthineers, Erlangen, Germany) with a slice thickness of 0.4 mm were employed to determine the dehiscences. To avoid selection bias and ensure rigorous criteria, only patients who presented at least one objective auditory fluctuation in pure-tone audiometry and/or those with a minimum of two episodes of spontaneous vertigo lasting longer than 20 min were selected for this test. These symptoms were used as they are considered reliable indicators of potential underlying pathologies that justify the use of advanced imaging techniques [1,8]. If these criteria were met, a 3T MRI (MAGNETOM Skyra, Siemens, Erlangen, Germany) was performed using a T2-FLAIR sequence for four hours after intravenous gadolinium administration to assess the presence of cochlear and vestibular EH.

### 2.4. Follow-Up

Clinical and audiovestibular function assessments were performed during the preoperative and post-operative periods. Symptom evolution was measured using a dichotomous variable: persistence or resolution of seven cardinal symptoms: unsteadiness, hearing loss, vertigo, tinnitus, autophony, the Hennebert (valsalva-induced vertigo) or Tullio phenomenon (sound-induced vertigo) [13], and falls. Hearing gain was evaluated by considering changes in pure tone averages, observing the most common pattern of hearing loss qualitatively. Vestibular function was assessed by recording VEMPs and vHIT results, categorized as normal or abnormal based on the criteria described above. Follow-up was conducted at the initial consultation, three months, and six months.

### 2.5. Medical Treatment

**Figure 1:**
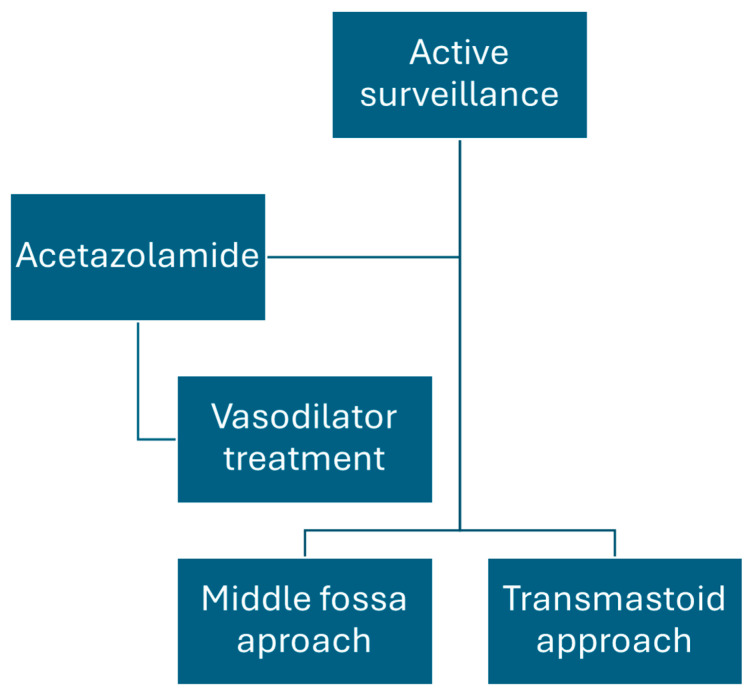
Summary of the therapeutic algorithm used in the patients in our sample.

The proposed treatment algorithm, which is outlined in Figure 1, was based on the severity of symptoms and their impact on quality of life. For most patients, active surveillance was deemed sufficient if they were asymptomatic or had mild symptoms. However, if symptoms significantly interfered with daily activities, the initial recommendation was to prescribe acetazolamide. If improvement was observed, the treatment was continued for an additional three months. In cases of poor tolerance, adverse effects, or inadequate response to acetazolamide, vasodilator therapy including betahistine, calcium channel blockers, or topiramate was performed as an alternative for the same duration. If these approaches were ineffective, surgical intervention was considered.

### 2.6. Surgical Treatment

**Figure 2:**
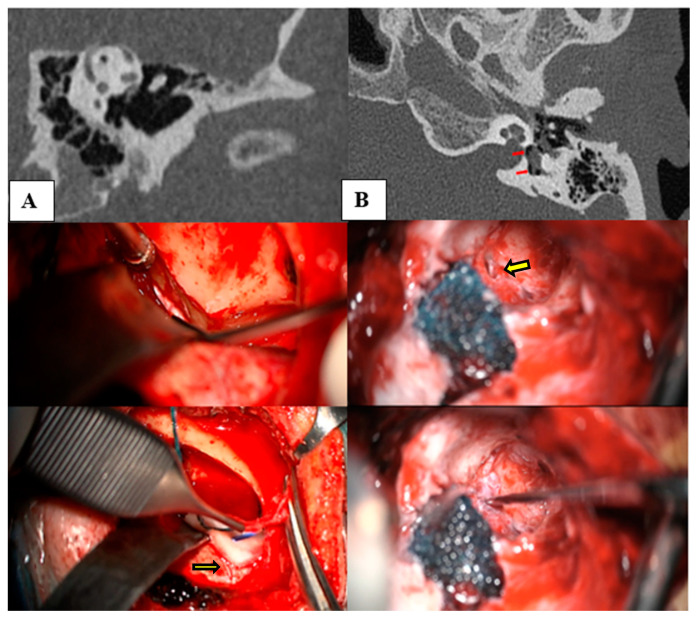
(**A**) (Left column) depicts the sequence of a middle fossa approach to cover the defect generated by a superior semicircular canal dehiscence (as seen in the CT scan). This approach includes the identification of the arcuate eminence, localization of the dehiscent canal (yellow arrow), and subsequent obliteration with autologous fascia. (**B**) (right column) depicts a retroauricular approach for sealing a perilymphatic fistula, which can be observed (red arrows in CT scan and yellow arrow intraoperative situation) with its levels of pneumolabyrinth in the vestibule, followed by its subsequent correction. Initially, autologous fascia was used, followed by reinforcement with autologous fat and Tissuecol.

Surgical treatment was employed for patients who experienced persistent symptoms significantly affecting their quality of life, despite medical management. For patients with SSCDS, the approach involved either a middle fossa or a transmastoid approach, obliterating the defect with autologous fascia as shown in Figure 2A. In patients with severe to profound hearing loss requiring an implantable device, the standard cochlear implant procedure was followed. For those with third window syndrome due to a perilymphatic fistula, a retroauricular approach was used to access the oval window, which was packed with autologous fat and fibrin glue (Tisseel; Baxter AG, Vienna, Austria), as shown in Figure 2B.

A significant number of patients also presented with bilateral involvement. In these cases, the decision of which ear to treat, if surgery was needed, was based on the results of audiovestibular tests—primarily VEMPs due to their high sensitivity in detecting these pathologies, along with otoneurological findings observed during the examination. The summary of the therapeutic algorithm is outlined in Figure 1.

### 2.7. Statistical Analysis

Descriptive statistical methods, including arithmetic means, standard deviations, and ranges, were employed for each group before and after treatment. A multivariate logistic regression analysis was conducted to evaluate the association between the presence of the seven cardinal symptoms at three different time points (pretreatment, at three months, and at six months). To measure the reduction in symptoms over time, the relative risk reduction (RRR) was calculated for each symptom, particularly comparing the pre-intervention period with the six-month follow-up. The 95% confidence intervals (CI) for the RRR values were reported to ensure the precision of the results.

A repeated measures ANOVA was used to assess the relationship between pre- and post-treatment pure tone average (PTA) and vestibular test results (VEMPs and vHIT), controlling for different treatments, sex, and patient age. The assumption of sphericity was verified using Mauchly’s test, and Greenhouse–Geisser corrections were applied if the assumption was violated.

Finally, to determine the association between the different groups in the cohort (SSCDS vs. other capsule dehiscences) and the development of EH, a chi-square test was performed. Subsequently, a logistic regression model was used to assess how various variables (age, gender, and duration of the disease) influenced the probability of developing EH. The normality of the data distribution was evaluated using the Shapiro–Wilk test. In cases where normality was not met, data transformations or alternative non-parametric tests were considered.

A *p*-value of <0.05 was considered indicative of statistical significance. Statistical analyses were performed using Stata version 16.1 (College Station, TX, USA).

## 3. Results

### 3.1. Population

A total of 86 patients were included in the study, and their demographic characteristics are summarized in Table 1.

### 3.2. Diagnosis and Associated Symptoms

The summary of the different diagnoses identified in our cohort, along with their characterization based on audiovestibular tests, is presented in Table 2.

From an audiometric perspective, 37.21% of the patients (n = 32) exhibited some form of hearing loss as documented by pure tone audiometry. The most common type of hearing loss was mixed, occurring in 62.5% of cases (n = 20), followed by conductive hearing loss in 28.13% (n = 9), and sensorineural hearing loss in 9.34% (n = 3). Furthermore, in three cases (3.49%)—two involving SSCDS and one involving a cochleo–carotid dehiscence—there was an absence of the stapedial reflex in a tympanogram. 

Regarding otoneurological findings, the most frequently observed type of nystagmus was torsional nystagmus elicited during hyperextension of the neck (41.67%), followed by horizontal nystagmus (33.33%), vertical downbeat nystagmus (12.5%), and vertical upbeat nystagmus (12.5%). Importantly, all cases of nystagmus were positional in nature, meaning they were non-specific in characteristics and lacked clear localizing value. They became evident only when visual fixation was suppressed. They did not follow a pattern of direction-changing nystagmus, and their intensity increased when a barometric change was induced using a Spiegel speculum (simulating the Hennebert phenomenon).

Notably, patients with SSCDS exhibited a significantly increased amplitude in ocular VEMPs with vibratory stimulus at 4 kHz, measuring up to 7.92 ± 15.37 dB HL. Other types of otic capsule dehiscences typically presented with an increased IAAR in oVEMPs, with a tendency towards reduced responses to acoustic stimuli. As can be inferred from Table 2, in the Other category, special findings of interest were noted for each subgroup.

### 3.3. Treatment

Regarding the approach taken, 34 patients (39.53%) did not receive medical treatment, either by personal choice or due to the mild nature of their symptoms. These patients were managed with active surveillance and the adoption of hygienic–dietary measures, such as avoiding sudden movements that could trigger vertigo episodes and minimizing exposure to loud noises and barometric changes that could exacerbate symptoms. However, among the patients with persistent symptoms that limited their daily lives, 42 (48.84%) received medical treatment. Of these, 23 (26.74%) completed treatment with acetazolamide. The remaining 19 patients (22.09%) were treated with vasodilator therapies. Lastly, 10 patients (11.62%) underwent surgical intervention. Among those with SSCDS, five were treated using a middle fossa approach, while one underwent a transmastoid strategy. The transmastoid approach was also employed for the two patients with perilymphatic fistula. The remaining two patients, one with an enlarged vestibular aqueduct and another with double dehiscence, both of whom had profound hearing loss in the affected ears, were managed with standard cochlear implantation surgery. Post-operative vertigo occurred in only four patients (40%), all of whom had SSCDS. This vertigo resolved within five days and was effectively managed with vestibular sedatives.

### 3.4. Follow-Up with Different Treatment Protocols

**Figure 3:**
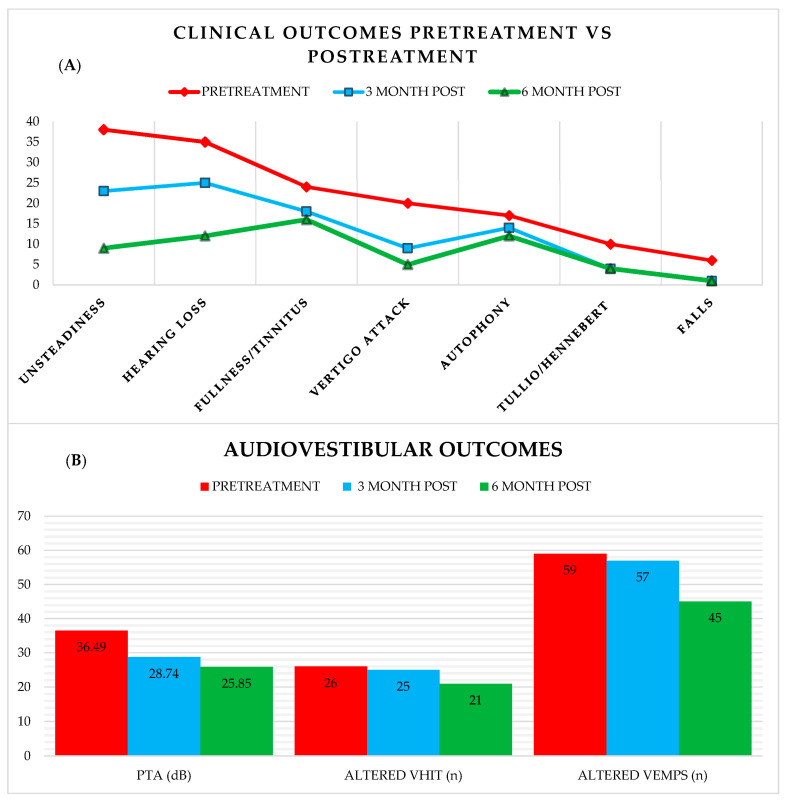
(**A**) shows the evolution of the 7 symptoms studied in our cohort at different follow-up times, while (**B**) shows the evolution of auditive and vestibular outcomes measured with pure tone audiometry for the auditive evaluation, vHIT and VEMPS for vestibular assessment.

Patient symptoms were assessed during the acute phase and subsequently at 3 and 6 months post-treatment, as shown in Table 3 and illustrated in Figure 3A,B.

As shown earlier in Table 3, all symptoms improved in terms of absolute reduction, with the most notable relative risk reduction (RRR) observed in cases of autophony, risk of falls, instability, and vertigo attacks, exceeding 75% with a CI of 95%. However, the statistical significance varied for each variable studied and depending on the treatment employed, as demonstrated in Table 4.

**Figure 4:**
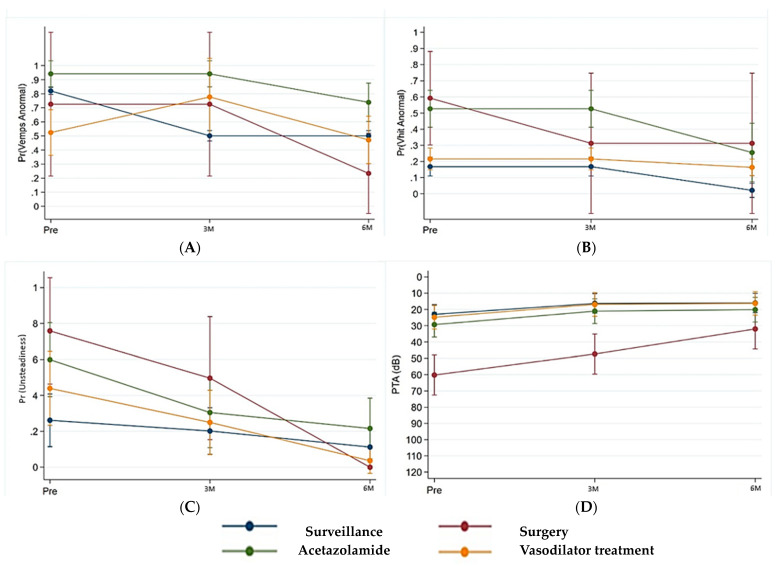
(**A**) (Top left) illustrates the evolution of the most frequent symptom found in our cohort—unsteadiness—comparing the different treatments considered in the study at the 3 follow-up time points. (**B**) (Top right) shows auditory evolution, while (**C**) (bottom left) and (**D**) (bottom right) assess vestibular function using VEMPS and vHIT, respectively. “Other” refers to other treatments used with a vasodilator effect.

The evolution of audiovestibular test findings at three and six months, as well as the follow-up of some of the most common symptoms in our sample with the different treatments considered, is represented in Figure 4.

### 3.5. Relationship to Possible Development of Radiologically EH

To explore the possible association between otic capsule dehiscences and the development of radiologically identifiable EH, only patients who met rigorous criteria—specifically, at least one objective auditory fluctuation in pure-tone audiometry and/or a minimum of two episodes of spontaneous vertigo lasting longer than 20 min—were selected for MRI using a T2-FLAIR sequence with gadolinium. A total of 16 (18.60%) patients out of the study cohort met these criteria and underwent this imaging test.

When comparing the incidence of EH between patients with SSCDS and those with other types of otic capsule dehiscences, it was found that 7.89% (n = 6) of the patients with SSCDS developed EH, compared to 40% of the patients with other otic capsule dehiscences (n = 4), a difference that was statistically significant (*p* = 0.0029).

Furthermore, using a logistic regression model to predict the probability of developing EH based on various variables, it was found that patient age was a significant predictor of risk, with a *p*-value of 0.042. However, gender did not reach statistical significance (*p* = 0.052), and the duration of the disease did not show a significant association with the development of EH (*p* = 0.798).

**Figure 5:**
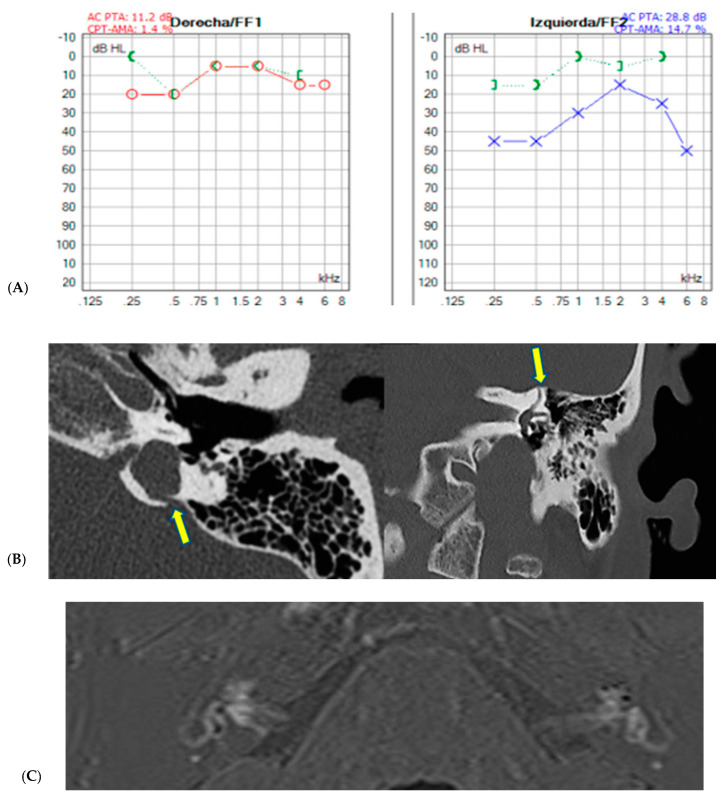
This is the case of a 46-year-old male who was diagnosed at another center with left-sided Ménière’s disease and had shown a poor response to intratympanic dexamethasone treatments. However, due to a clear Hennebert’s phenomenon and the presence of a type B tympanogram with preserved stapedial reflexes in the context of conductive hearing loss, as shown in (**A**). Both bone conduction showed in green and red and blue representing air conduction. A temporal bone CT scan was performed (**B**), revealing a double dehiscence at the level of the vestibular aqueduct, involving the jugular vein and the superior semicircular canal (yellow arrows). Additionally, as observed in (**C**) on the real IR sequence, a hypointensity signal was found in the cochlea, compatible with endolymphatic dilatation at that level (signs of moderate cochlear hydrops in the left ear).

**Figure 6:**
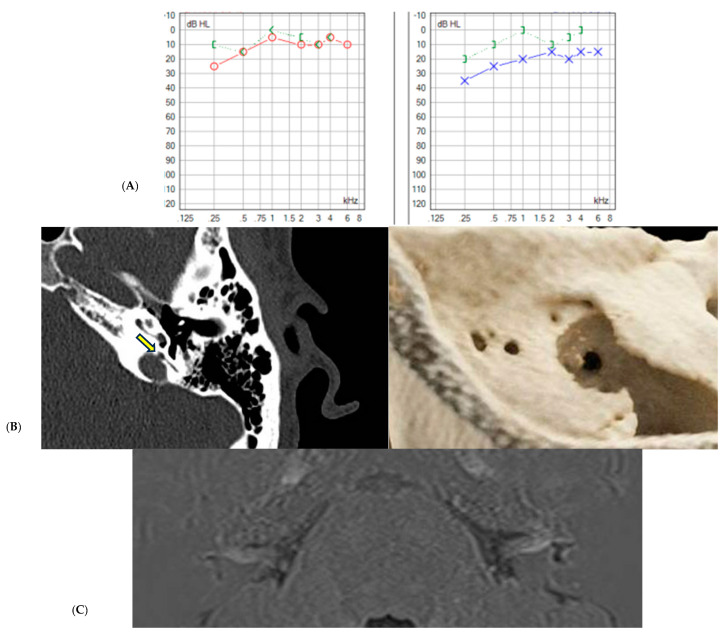
This is the case of an 18-year-old male who presented with loss of consciousness following Tullio and Hennebert phenomena, preceded by otic symptoms and fluctuations in the left ear, as shown in (**A**), with both bone conduction showed in green and red and blue representing air conduction. A temporal bone CT scan revealed third window syndrome at the level of the ampulla of the posterior semicircular canal involving the jugular bulb, which was later confirmed in a 3D reconstruction, as shown in (**B**) with yellow arrow. Due to recurrent episodes of vertigo, along with up to three subsequent episodes of fluctuations, an MRI with hydrops sequences (**C**) was eventually requested, showing dilation of the saccule, confluent with the utricle, indicative of moderate left vestibular hydrops.

Finally, the multiple findings discussed in this study are illustrated with two examples summarized below in Figure 5 and Figure 6.

## 4. Discussion

Third mobile window syndromes may originate embryologically due to an alteration in the development of the otic capsule or due to acquired causes [15,16,17]. This concept has emerged as a significant clinical diagnosis and appears to be more common than previously thought. Consideration of new entities beyond the three classic third mobile window etiologies outlined in the 2015 Bárány Society consensus [1] has not only increased their incidence but also expanded the range of symptoms they produce [16].

From a clinical perspective, this study demonstrates that the symptoms associated with these entities extend beyond the typical presentation of otolithic dysfunction caused by sound and pressure (Tullio or Hennebert phenomena) as well as autophony. They may even contribute to cognitive impairments, loss of spatial orientation, or anxiety manifestations due to the instability they cause, which is the most found symptom. Several studies [7,18,19] have reported that approximately 75% of patients experienced mixed hearing loss with an air-bone gap, which is consistent with our sample, whereas about 64% exhibited this audiometric deficit, particularly at low frequencies (250–500 Hz).

Furthermore, the increased incidence and description of new etiologies acting as third mobile window patterns have improved our understanding of otolithic organ physiology through detailed vestibular testing, mainly VEMPs [20]. In 2020, Tran et al. [21] and more recently Curthoys et al. [22] observed that acoustic stimuli in cervical VEMPs could act as inhibitory stimuli, especially at 250 and 500 Hz, resulting in reduced thresholds but increased amplitude. By contrast, these stimuli behaved excitatorily in ocular VEMPs, with similar thresholds but significantly higher amplitudes, especially at high frequencies like 4000 Hz. These findings are relevant compared to other third window syndrome etiologies, such as perilymphatic fistulas (Hermann and Coelho, 2014 [23]), where decreased sensitivity to acoustic stimuli is more common due to the hydropic sac exerting pressure on the stapes. In line with the literature [24], our cohort also showed alterations in VEMPs among patients with SSCDS, presenting as increased ocular amplitude with vibratory stimulation (7.92 ± 15.37 dB HL) before treatment.

Therapeutically, it is estimated that about half of the patients diagnosed with SSCDS, the most common condition in our cohort at over 88%, opt for an intervention to alleviate symptoms, while the other half find sufficient reassurance in understanding the cause of their symptoms [25]. However, our study also explores the possibility of less invasive treatments than surgery, whereby the most used medications were diuretics such as acetazolamide and subsequently vasodilator drugs. The use of these treatments in the study was empirical, as the efficacy of these drugs for this condition had not been established. The plausible hypothesis for their potential efficacy is that diuretic or vasodilator effects might reduce pressure gradients and alter inner ear homeostasis, thereby alleviating symptoms arising from abnormal fluid dynamics in these cochleo–vestibular capsule abnormalities [26,27,28]. This theory is supported by our cohort’s results, particularly with diuretics, where symptom reduction was observed with statistically significant results for symptoms such as instability, vertigo attacks, and tinnitus, as well as for objective measures such as PTA and VEMPs.

It is essential to emphasize the importance of differentiating otic capsule dehiscences from Ménière’s disease, as both conditions can present with overlapping symptoms, including vertigo, hearing loss, and tinnitus [1]. When Prosper Ménière first described the disease in 1861 as a constellation of symptoms characterized by episodic vertigo, fluctuating hearing loss, tinnitus, and aural fullness, he could not have anticipated the discovery of entities such as otic capsule dehiscences, among others, that can closely mimic the clinical presentation of Ménière’s disease [29]. For this reason, it is essential to adhere strictly to diagnostic criteria while remaining mindful of other conditions that can simulate it. Failure to differentiate these disorders can result in misdiagnosis and delays in appropriate management, emphasizing the need for thorough clinical evaluation and targeted imaging studies to accurately identify otic capsule dehiscences.

Today, there is no doubt that the only truly curative treatment for third window syndromes is surgery [29,30,31], whether through a middle fossa approach, which is more common, a transmastoid approach, or even working in the round window area. In our cohort, where a considerable number underwent surgery (around 11%), the results align with the literature, contributing to the improvement of symptoms and normalization of audiovestibular tests (as shown in Figure 3), with all results achieving statistical significance except for autophony. Another interesting finding, although not precisely quantified in the results, is that in the post-operative group, patients who experienced hearing improvement showed not only enhanced PTA thresholds: in most cases of mixed or conductive hearing loss, an early closure of the air–bone gap was observed.

Finally, this study’s uniqueness lies in exploring the potential relationship between third window phenomena and radiologically observable EH. To date, most studies [32,33,34] have focused on establishing a relationship between dilated vestibular aqueduct and EH. However, it remains unclear whether third window phenomena precede the disease or result from it. Recent studies have significantly correlated both findings, reinforcing the theory that temporal bone anatomical variations can alter intralabyrinthine fluid dynamics. Our cohort’s results indicate that the risk of developing EH is higher in patients with otic capsule dehiscences other than SSCDS, with statistical significance. Considering that EH is believed to begin in the cochlea’s basal turn, possibly progressing along the basal spiral and eventually affecting the vestibule [35,36,37], the proximity of dehiscent areas to these points where EH typically originates could explain the higher proportion of hydropic disease in cases where the dehiscent area is not located along the superior semicircular canal. This hypothesis provides an important basis for future research in this field, encouraging large-scale comparisons and further investigations with dynamic MRI studies in EH sequences to better understand this relationship.

Given the heterogeneity of the patient population, the selection criteria were designed to focus on those with the most indicative symptoms of potential EH. Although not all patients underwent MRI, the chosen subset represents those most likely to present with radiologically detectable EH, which strengthens the relevance of the findings within this group. While the absence of universally applied diagnostic measurements is noted, the study’s comparative analysis and use of logistic regression help to control for these variables.

### Limitations

The main limitation of this study primarily concerns the follow-up duration. While the sample size is significant given the low incidence of this condition, extending follow-up beyond six months could yield more robust conclusions and potentially greater statistical significance and relevance. However, it is important to consider that the medical treatments used, in particular, can produce multiple adverse effects and hydroelectrolytic imbalances, making long-term treatment maintenance inadvisable.

Another major limitation of this study is the lack of a specific standardized questionnaire to assess quality of life in patients with third window syndromes, such as SSCDS and other otic capsule dehiscences. General tools such as the dizziness handicap inventory (DHI) and Glasgow benefit inventory were not used, because these patients often have non-specific and variable symptoms, with only a third experiencing clear vertigo attacks. This absence limits the consistent measurement of the disorders’ impact. Instead, we focused on applying patient-reported outcome measures (PROMs), as described throughout our work, to monitor their progress and evaluate their evolution. However, there is a need for developing precise tools to better understand and assess these patients’ challenges.

To conclude, to study the association with EH, the rigorous selection criteria employed in this study were intended to minimize selection bias and ensure that only the most clinically relevant cases were analyzed. However, this approach may limit the generalizability of the findings to a broader population. Future studies could explore the utility of these criteria in more diverse patient groups. Further research is needed to fully understand these entities, including longitudinal studies with extended follow-up. Additionally, it is essential to include in vivo functional neuroimaging studies to better explore the pathophysiology of these dehiscences and their potential relationship to the risk of developing inner ear hydrops.

## 5. Conclusions

Otic capsule dehiscences are clinically significant conditions that are often underdiagnosed due to a lack of clinical suspicion. This study underscores the importance of considering these dehiscences, as well as third window syndrome, in patients presenting with symptoms similar to Ménière’s disease, particularly when there are discrepancies in audiovestibular test results.

Despite the utility of tests like VEMPs, which are highly sensitive and specific, diagnostic confirmation should rely on detailed imaging studies, such as high-resolution temporal bone CT or cone beam CT. These tools allow for precise localization and characterization of otic capsule defects responsible for clinical symptoms.

Although the strict selection criteria and focused population may limit the generalizability of our findings, they were essential for ensuring the reliability and clinical relevance of the observed associations. Future research should aim to validate these findings in broader and more diverse patient cohorts, with an emphasis on extended follow-up and advanced neuroimaging techniques.

## Figures and Tables

**Table 1:** Summary of demographic data in our study.

Demographic Description			
Age at Diagnosis	52.2 ± 7.64 (6–78) years		
Gender	54 (62.76%) female/32 (37.21%) male		
Disease Evolution	3.83 ± 1.12 years (6 months–5.6 years)		
Ear	8 (9.30%) bilateral	36 (41.86%) right side	42 (48.83%) left side

**Table 2:** Summary of clinical, auditory, and vestibular findings and possible radiologically observable development of EH in the studied patients. Three subgroups within the third mobile window syndrome have been included according to the criteria of López-Escámez et al. [1], while the different types of otic capsule dehiscence (Type I, III, and double dehiscences) have been classified based on the description by Reynard et al. [14].

Diagnosis		n	%	Symptoms	PTA (dB)	vHIT Altered	VEMPs Altered	EH	Other
Third window syndrome 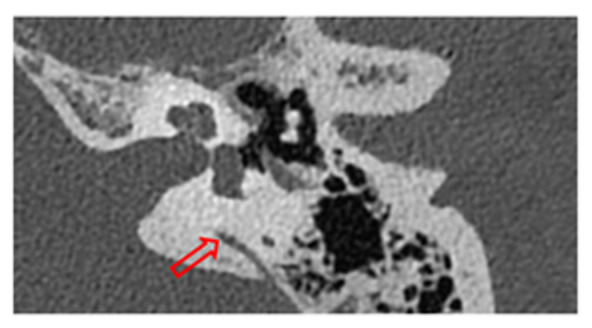	Type I (SSCD)	76	88.37%	Unsteadiness: 34.88% Hearing loss: 32.56% Fullness/tinnitus: 22.09% Vertigo attack: 19.76% Autophony: 16.27% Tullio/Hennebert: 9.30% Falls: 5.81%	28 dB	Yes (n = 24)	Yes (n = 55)	Yes (n = 6)	Increased oVEMP amplitude at 4 kHz
	Perilymphatic fistula	2	2.32%		105 dB	Yes (n = 2)	Yes (n = 2)	No	+ Fistula sign with Siegel’s speculum
	Enlarged vestibular acueduct	2	2.32%		95 dB	Yes (n = 2)	Yes (n = 2)	No	Non syndromic association
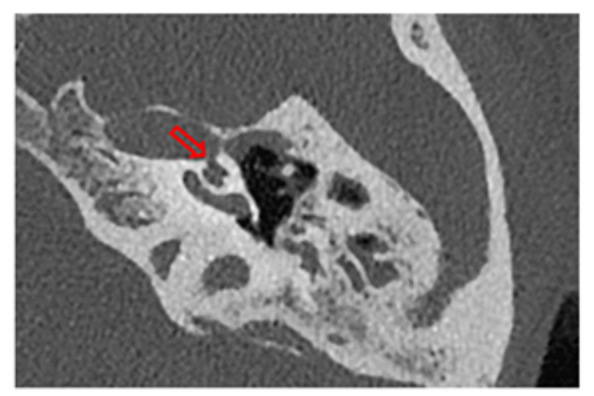 Other otic capsule dehiscences	Type II Cochleo–carotid and PSC–jugular vein	2	2.32%	Hearing loss: 100% Autophony: 66.67% Unsteadiness: 50% Vertigo attack: 33.33% Fullness/tinnitus: 16.67% Tullio/Hennebert: 16.67% Falls: 16.67%	52 dB	No	No	Yes (n = 1)	Type B timpanogram with absent stapedial reflex
	Type III Cochleo–facial and Cochleo-ICA	2	2.32%		103 dB	Yes (n = 1)	Yes (n = 1)	Yes (n = 1)	Gusher during cochlear implant
	Double dehiscences (Cochleo–ICA + Cochleo–facial) and SSCD + jugular vein-EVA	2	2.32%		59 dB	Yes (n = 1)	Yes (n = 1)	Yes (n = 2)	Gusher during cochlear implant

**Table 3:** Summary and representation of clinical and audiovestibular outcomes pre- and post-treatment. RRR: relative risk reduction. CI: confidence interval.

Symptom/Measure	Pretreatment	3 Month Post	6 Month Post	RRR (1–6 Months) or Gain	CI 95%
Unsteadiness	38 (44.19%)	23 (26.74%)	9 (10.47%)	76.30%	[62.80–89.83%]
Hearing loss	35 (40.17%)	25 (29.07%)	12 (13.95%)	65.97%	[49.33–81.44%]
Fullness/tinnitus	24 (27.91%)	18 (20.93%)	16 (18.60%)	33.29%	[14.47–52.19%]
Vertigo attack	20 (23.26%)	9 (10.47%)	5 (5.81%)	75.03%	[56.02–93.98%]
Autophony	17 (19.77%)	14 (16.28%)	12 (13.95%)	88.24%	[72.92–100.00%]
Tullio/Hennebert	10 (11.63%)	4 (4.65%)	4 (4.65%)	60.14%	[29.64–90.36%]
Falls	6 (6.98%)	1 (1.16%)	1 (1.16%)	83.33%	[53.51–100.00%]
PTA (dB)	36.49	28.74	25.85	10.64 dB	-
VEMPs altered (n)	59 (68.60%)	57 (66.27%)	45 (52.32%)	23.73%	[12.87–34.58%]
vHIT altered (n)	26 (30.23%)	25 (29.07%)	21 (24.41%)	19.23%%	[4.08–34.38%]

**Table 4:** Summary of *p*-values for each symptom, depending on each treatment, comparing the pre-intervention period with the post-intervention period after 6 months. *p*-values marked with an asterisk (*) indicate statistical significance for *p* < 0.05.

Treatment/Symptoms	Active Surveillance (n = 34)		Acetazolamide (n = 23)		Vasodilator Treatment (n = 19)		Surgery (n = 10)	
Unsteadiness	*p* = 0.355		*p* = 0.018 *		*p* = 0.033 *		*p* = 0.001 *	
Hearing loss	*p* = 0.099		*p* = 0.283		*p* = 0.082		*p* = 0.003 *	
Fullness/tinnitus	*p* = 0.721		*p* = 0.009 *		*p* = 0.316		*p* = 0.001 *	
Vertigo	*p* = 0.084		*p* = 0.001 *		*p* = 0.082		*p* = 0.001 *	
Autophony	*p* = 0.422		*p* = 0.282		*p* = 0.336		*p* = 0.331	
Tulio/Hennebert sign	*p* = 0.744		*p* = 0.188		*p* = 0.689		*P* = 0.028 *	
Falls	*p* = 0.071		*p* = 0.052		*p* = 0.065		*p* = 0.038 *	
PTA	*p* = 0.165		*p* = 0.001 *		*p* = 0.144		*p* = 0.015 *	
Vestibular tests	vHIT	*p* = 0.366	vHIT	*p* = 0.098	vHIT	*p* = 0.152	vHIT	*p* = 0.082
	VEMPS	*p* = 0.525	VEMPS	*p* = 0.005 *	VEMPS	*p* = 0.033 *	VEMPS	*p* = 0.008 *

## Data Availability

Data pertaining to this study can be shared upon request to the corresponding author.
